# Radiomics-based MRI models for predicting breast cancer axillary lymph node involvement in comparison with Node-RADS: a proof-of-concept study

**DOI:** 10.1186/s41747-025-00660-4

**Published:** 2025-12-02

**Authors:** Roberto Maroncelli, Veronica Rizzo, Marcella Pasculli, Sara Coppola, Chiara De Nardo, Marco Moschetta, Carlo Catalano, Federica Pediconi

**Affiliations:** 1 Department of Radiological, Oncological and Pathological Sciences, Sapienza—University of Rome, Rome, Italy; 2 Department of Experimental Medicine, Sapienza—University of Rome, Rome, Italy; 3 Interdisciplinary Department of Medicine (DIM), Section of Radiology and Radiation Oncology, University of Bari, Bari, Italy

**Keywords:** Breast neoplasms, Lymph node metastases, Machine learning, Magnetic resonance imaging, Node-RADS

## Abstract

**Background:**

Detection of axillary lymph node (LN) involvement is essential for staging breast cancer and optimizing treatment. This proof-of-concept two-center study explored the feasibility of magnetic resonance imaging (MRI) radiomics-based machine learning models to predict LN involvement and compare their performance with node reporting and data system (Node-RADS).

**Materials and methods:**

We retrospectively included breast cancer patients undergoing preoperative multiparametric MRI and LN dissection (January 2020–September 2024). Stable radiomic features (intraclass correlation coefficient ≥ 0.75) were extracted from contrast-enhanced, subtracted, and T2-weighted sequences. Five machine learning models were trained for binary LN involvement classification, using histopathology as a reference standard. The best-performing model was externally validated on an independent cohort. Performance metrics included sensitivity, specificity, positive predictive value (PPV), negative predictive value (NPV), and area under the receiver operating characteristic curve (AUROC). Node-RADS (scores > 2 indicating LN involvement) was used for comparison in the external dataset.

**Results:**

Of 93 cases, 40 (43%) were LN involvement-positive; 17 stable features were selected for model development. The best-performing model achieved 81% AUROC (95% confidence interval 78–85%), 75% accuracy (70–79%), 52% sensitivity (41–62%), 92% specificity (86–98%), 85% PPV (76–95%), and 72% NPV (68–76%) on the internal dataset. External validation (18 cases) showed promising results: 94% AUROC (89−99%), 89% sensitivity (52–100%), 100% specificity (66–100%); in this small cohort, accuracy, sensitivity, and specificity did not differ significantly *versus* Node-RADS, with moderate agreement (Cohen κ = 0.47).

**Conclusion:**

In this preliminary series, the model showed performance metrics in predicting LN involvement comparable to Node-RADS.

**Relevance statement:**

Radiomics-based MRI models may represent a promising investigational tool for noninvasive axillary LN assessment in breast cancer. The performance comparable to Node-RADS suggests a potential to support clinical decision-making in the context of axillary de-escalation surgery.

**Key Points:**

- Radiomics uses MRI to predict breast cancer LN involvement non-invasively and accurately.
- Radiomics and Node-RADS showed comparable performance.
- Radiomics could reduce invasive procedures, supporting personalized treatments in breast cancer care.

**Graphical Abstract:**

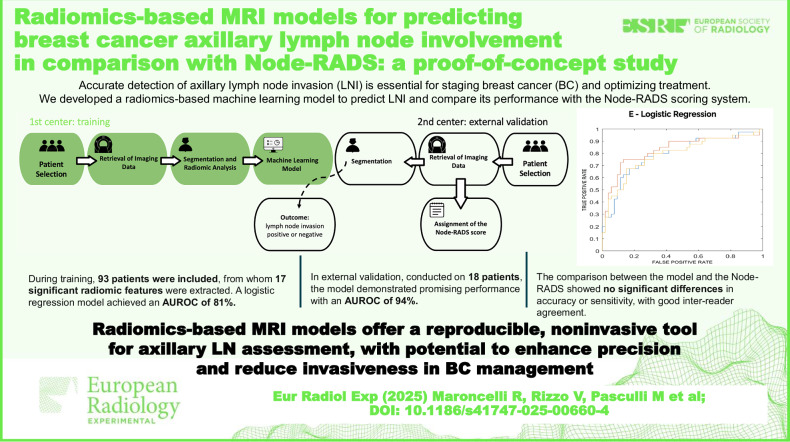

## Background

Axillary lymph node (LN) involvement remains a critical factor in determining breast cancer management strategies, influencing both surgical decisions and systemic treatments [1−3]. Despite advancements, current preoperative assessments often rely on techniques like sentinel LN biopsy [2] or imaging-based evaluations, including Node Reporting and Data System (Node-RADS). This system offers reliable yet sometimes subjective insights into LN status [4, 5]. These approaches are further challenged by their invasiveness or dependence on qualitative interpretation, potentially leading to variations in clinical outcomes [6, 7].

Radiomics, with its capacity for quantitative feature extraction from routine imaging modalities, introduces a promising, transformative approach to LN involvement prediction [8]. This methodology, coupled with machine learning, has shown promise in enhancing diagnostic precision across various cancers by identifying imaging biomarkers that are often imperceptible to human observers [9]. Applications in breast magnetic resonance imaging (MRI) have specifically highlighted its potential for noninvasive LN evaluation, demonstrating high accuracy and predictive value when combined with robust computational algorithms [10].

Building on this foundation, our study aimed to develop and validate a radiomics-based machine learning model using multiparametric MRI to predict LN involvement in breast cancer patients. By comparing the model’s diagnostic performance to the Node-RADS classification system, we aimed to explore its potential utility as an investigational tool for noninvasive preoperative LN assessment. This proof-of-concept study provides preliminary insights into the feasibility of integrating radiomics into clinical workflows for axillary LN assessment in breast cancer. Using histopathology as the reference standard, it explores the potential of radiomics to complement radiologist interpretations and highlights the need for noninvasive, reproducible, and precise tools to support personalized treatment planning.

## Materials and methods

### Study design and patient population

This two-center retrospective study was approved by the Institutional Review Boards of both participating centers, with informed consent requirements waived. All patient data was handled in accordance with relevant data protection regulations and institutional review board guidelines. This study was designed, conducted, and reported in adherence to the Checklist for Clear Reporting of Radiomics Studies to ensure methodological rigor and transparency [11, 12].

Patients diagnosed with invasive carcinoma, microinvasive carcinoma, or high-risk (grade 3, high Ki-67) carcinoma *in situ* [13] between January 2020 and September 2024 were retrospectively enrolled. Eligible patients underwent preoperative multiparametric MRI, breast surgery, and axillary LN dissection at either center following identical selection criteria.

Patients with positive LNs on imaging indicating locally advanced disease (*e.g*., stage II or higher) or those undergoing axillary LN dissection due to sentinel LN failure, cT4 tumors, or inflammatory carcinoma were included.

Exclusion criteria included prior neoadjuvant systemic therapy, incomplete MRI protocol, absence of documented LN pathology examination, and missing clinical or imaging data. All cases included had complete imaging, clinical, and histopathological data, ensuring robust data integrity for model training and validation.

The sample size of this study was limited by the decision to exclude patients who had undergone neoadjuvant therapy, ensuring a direct correlation between the Node-RADS score and the histopathological analysis of LNs without the potential morphological alterations induced by treatment. Although this selection significantly reduced the number of eligible patients, it was necessary to maintain methodological consistency with the validation criteria previously established for Node-RADS, as reported in a prior 2024 study by Pediconi et al [5].

To evaluate and compare the performance of the radiomics-based model and the radiologist’s assessment, only the cases from the external validation dataset were used. Cases from the first center were allocated exclusively to the development and validation of the radiomics model, ensuring an unbiased comparison of the radiologist’s Node-RADS scoring and model performance. This approach ensured that cases used to develop and validate the model did not overlap with those used for the comparison, maintaining methodological rigor.

For each patient, we considered the highest Node-RADS score assigned by the radiologist (F.P.) and then matched against the final pathology report to determine the presence or absence of LN involvement. Subsequently, the same LN with the highest Node-RADS score was segmented using the software to ensure consistency and comparability between the radiologist-assigned score and the radiomics analysis.

**Fig. 1:**
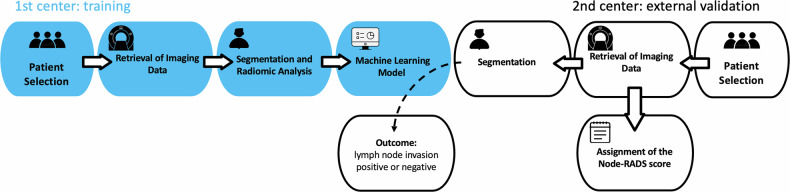
Study workflow. From left to right, the workflow illustrates the development of the machine-learning model using data collected from the first center. From right to left, the workflow outlines the validation of the model using data collected from the second center. The same radiologist who segmented both datasets assigned the Node-RADS scores to the cases used for external validation

The workflow diagram summarizing the process is presented in Fig. 1.

### MRI protocol

Breast MRI was performed at both centers using scanners equipped with an 8-channel dedicated breast coil: a 3-T Discovery MR 750 (GE Healthcare) at Center 1 in Rome, Italy; and a 1.5-T Achieva (Philips Medical Systems) at Center 2, Bari, Italy. A uniform imaging protocol was applied, including axial T2-weighted fat-suppressed sequences, diffusion-weighted imaging (*b*-values 0, 500, and 1,000 s/mm²), apparent diffusion coefficient maps, and dynamic contrast-enhanced T1-weighted fat-suppressed sequences acquired before and after gadolinium injection. Subtraction images were generated for all examinations.

For the radiomics analysis, only the axial T2-weighted and the venous phase post-contrast T1-weighted subtracted sequence (90 s after contrast injection) were selected for further evaluation. The technical parameters of these two sequences are described in detail in Table 1.

**Table 1:** Technical parameters of the T2-weighted and contrast-enhanced T1-weighted dynamic sequences

Sequences	Technical characteristics	
	Center 1	Center 2
Fast spin-echo T2-weighted		
Repetition time (ms)	9,000–11,000	6,300
Echo time (ms)	119–120	130
Acquisition matrix	512 × 224	600 × 336
Slice thickness (mm)	3–5	3
Field of view	350 × 350	250 × 450
Number of excitations	1	1
Scan time (s)	130	130
Axial T1-weighted fat-saturated		
Flip angle (°)	15	15
Repetition time (ms)	8	4
Echo time (ms)	4	2
Acquisition matrix	512 × 256	300 × 168
Slice thickness	1.40	1.60
Field of view	380 × 380	250 × 450
Number of excitations	1	1
Scan time (s)	210 (42 per acquisition)	540 (90 per acquisition)

### Radiomic analysis

**Fig. 2:**
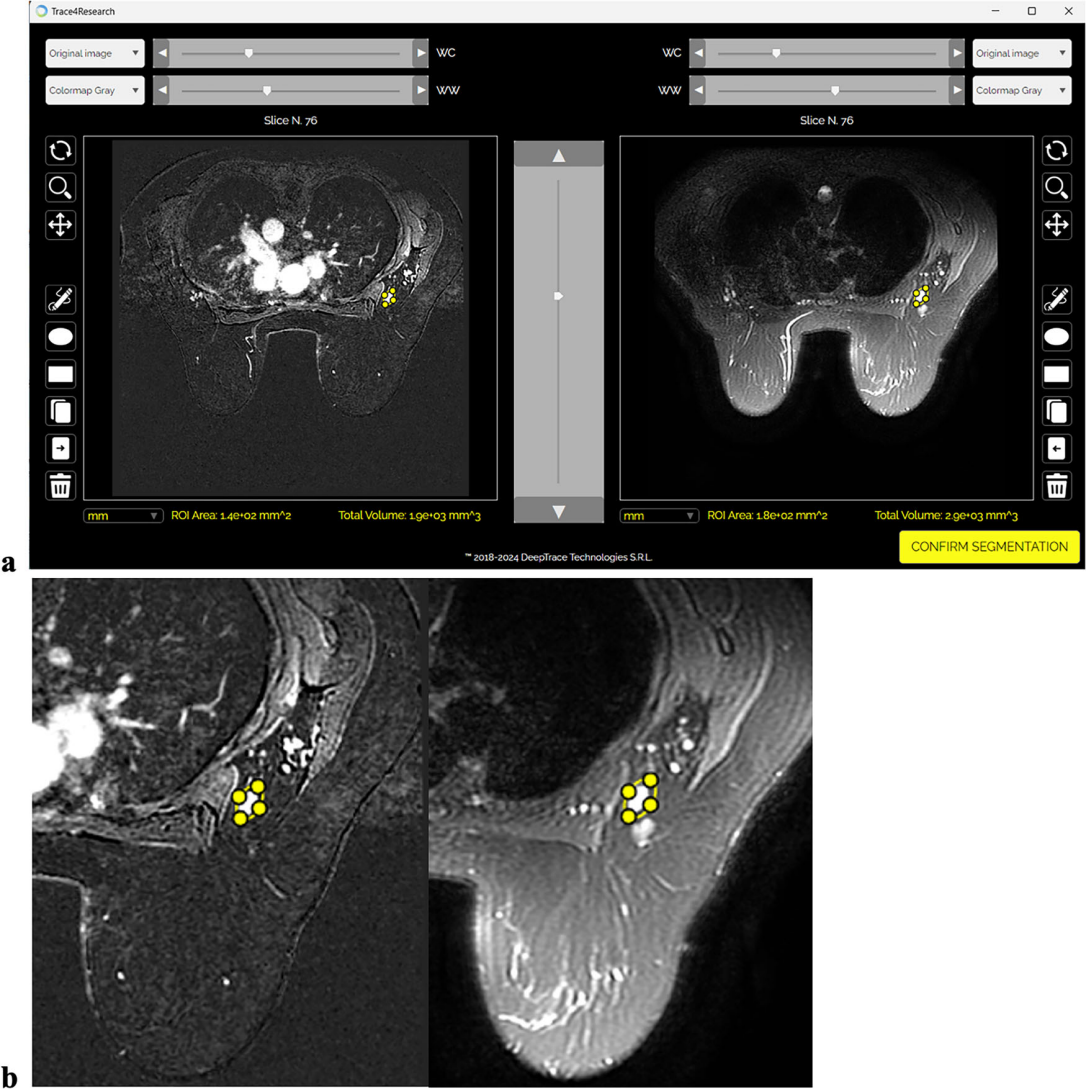
TRACE4Research™ platform: segmentation panel showing the contrast-enhanced subtracted images on the left and the T2-weighted images on the right (**a**); magnified left breast and axilla with node segmentation on both sequences (**b**)

Radiomic analysis was performed in compliance with the image biomarker standardization initiative (IBSI) [14] using the TRACE4Research™ platform (DeepTrace technologies) [15]. Automated workflows included segmentation, preprocessing, feature extraction, and selection. LNs were manually segmented slice-by-slice by a radiologist (F.P.) with 20 years of experience (Fig. 2). Images were resampled to isotropic voxel spacing (1 mm for subtraction images, 2 mm for T2-weighted sequences). Extracted radiomic features included morphology, intensity-based statistics, intensity histograms, and texture features (GLCM, GLRLM, GLSZM, NGTDM, and NGLDM), derived from original and filtered images (wavelet, square, squareroot, logarithm, exponential, gradient, and laplacian-of-gaussian).

Radiomic feature selection was performed using a multistep approach, initially applying a reproducibility assessment based on the intraclass correlation coefficient (ICC) to retain stable features. Feature stability and repeatability were quantitatively assessed using the ICC, with a threshold of ≥ 0.75 considered acceptable for inclusion, in line with IBSI recommendations. Features below this threshold were excluded.

Subsequently, a mutual-information analysis and a genetic algorithm were applied for feature selection, using a custom fitness function that maximized relevance (mean mutual information with the class label, using symmetric uncertainty) and minimized redundancy (mean inter-feature correlation). Discretization was performed using 12 bins. This process was executed within each fold of a nested 3-fold cross-validation to avoid data leakage, ensuring that selection and training occurred independently of validation or testing data.

Features with a low coefficient of variation (threshold = 0.1) and low mutual information with the class label (threshold = 0.3) were removed. To address the issue of intercorrelated features, a mutual-information analysis was conducted, using a genetic algorithm to optimize a custom fitness function. This function operates on a selected set of candidate features and considers two factors: the mean intercorrelation, expressed as symmetric uncertainty among the discretized features (12 bins), and the mean correlation of each feature with the class label, also expressed as symmetric uncertainty. Therefore, the resulting set of features is chosen to maximize useful information and minimize redundancy.

To prevent data leakage, feature selection was performed independently within each fold of the nested 3-fold cross-validation, ensuring that no information from validation or test sets influenced the training process. This process was repeated independently in each fold, ensuring that feature selection was based solely on the training data of that specific fold, without access to the validation or test sets.

To address the issue of segmentation variability, we evaluated the stability of the radiomic features with respect to different segmentations and their repeatability in a test-retest study. This evaluation was performed by statistically comparing the features obtained from data augmentation strategies: (a) randomly manipulating the manual segmentation of the volume of interest performed by the expert operator; and (b) rotating the original images and segmentations. The comparison was performed by computing the ICC; features with ICC < 0.75 were considered non-stable/repeatable.

Five machine-learning models were tested: random forest, support vector machine, k-nearest neighbors, multilayer perceptron, and logistic regression. Models were trained and validated using nested 3-fold cross-validation, with oversampling of the minority class using “adaptive synthetic sampling”.

Performance metrics were averaged across validation folds with 95% confidence intervals and significance assessed using the Wilcoxon signed-rank test. The best-performing model, based on mean area under the receiver operating characteristic curve (AUROC) from internal testing, underwent external validation using an independent dataset.

### Node-RADS assessment

**Fig. 3:**
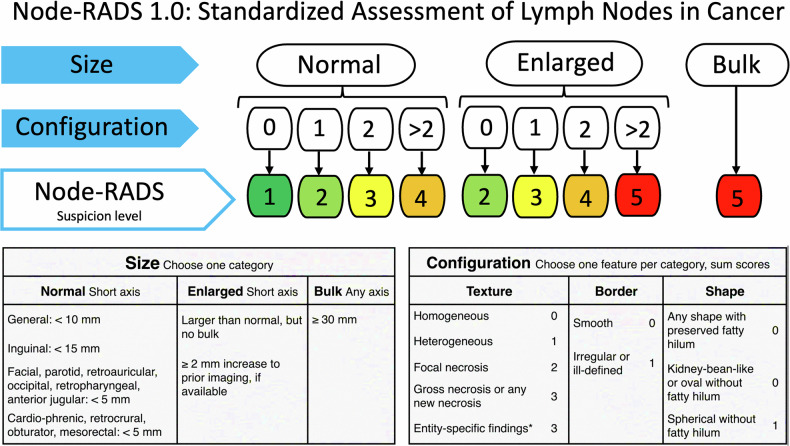
Explanation of the Node-RADS scoring system, adapted from the original publication [4]. The configuration score is calculated by summing points from three subcategories: texture, border, and shape. This combined configuration score is then integrated with the size evaluation using the flowchart to assign a final Node-RADS score (from 1 to 5), which reflects the likelihood of malignancy

The same breast radiologist (F.P.) who segmented both datasets evaluated LNs using the Node-RADS scoring system [4, 5] (Fig. 3), blinded to histopathological results. Scores ranged from 1 (very low suspicion) to 5 (very high suspicion). LNs rated as Node-RADS > 2 were classified as positive for LN involvement [5].

### Histopathological analysis

Lymph-node status was confirmed via axillary LN dissection. LNs were classified as negative, with micrometastasis (> 0.2 mm and ≤ 2 mm), or with micrometastasis (> 2 mm). Pathological results served as the reference standard for assessing LN status, with findings confirming the classification. Cases of isolated tumor cells (< 0.2 mm) were classified as benign [16].

### Statistical analysis

Statistical analysis was performed using embedded tools within the TRACE4Research™ platform. Radiomic feature distributions for LN involvement-positive and LN involvement-negative groups were analyzed using violin and box plots. Non-parametric univariate Wilcoxon rank-sum tests evaluated the discriminatory power of radiomic features, with Bonferroni–Holm adjustment for multiple comparisons.

Model performance was compared with Node-RADS using the McNemar test for paired sensitivity, specificity, and accuracy, and Cohen κ for agreement. Metrics were presented with 95% confidence intervals. External validation results were included to confirm the robustness and generalizability of the machine learning model.

A *p*-value < 0.05 was considered statistically significant.

## Results

### Datasets

**Fig. 4:**
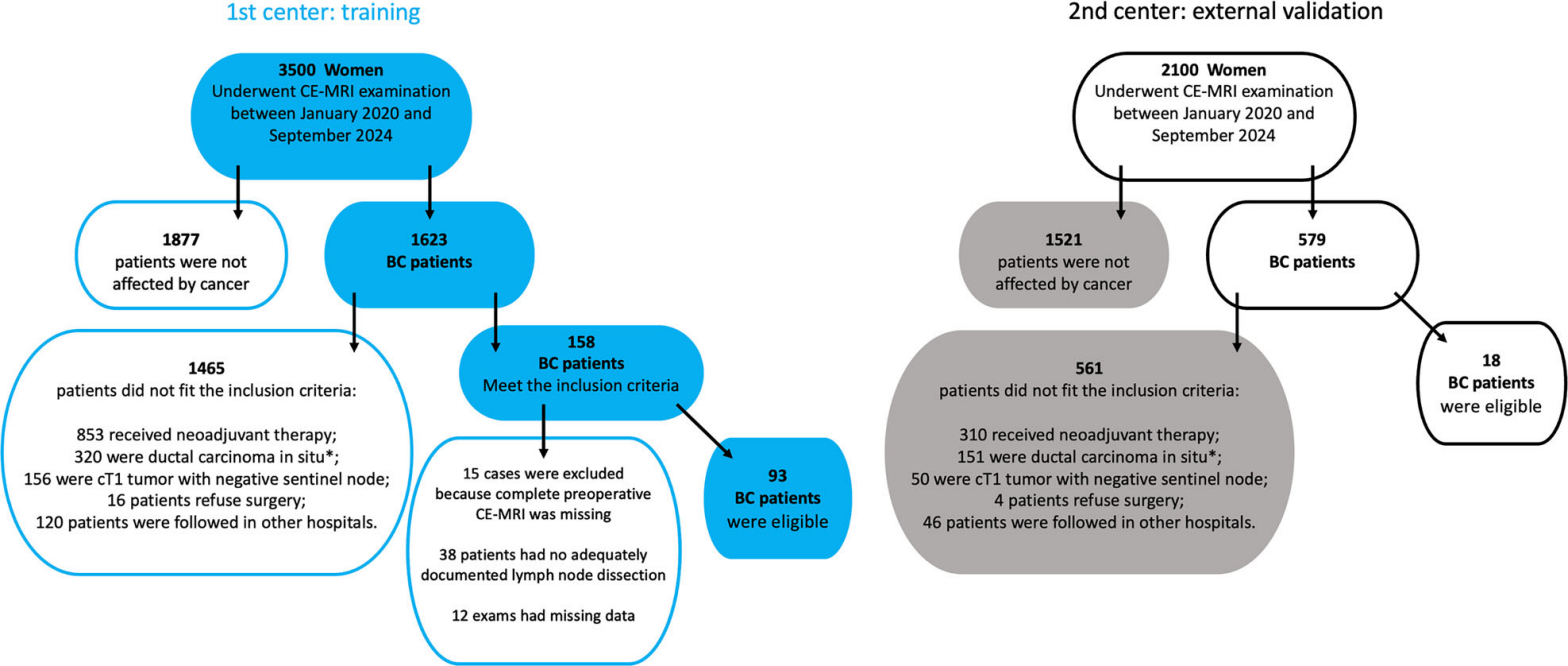
Flowchart of participants: on the left, the first center, on the right, the second center. ^*^High-risk ductal carcinoma *in situ* (G3, high Ki67) was included in the study

From a database of 3,500 women who underwent MP-MRI examination between January 2020 and September 2024, 1,623 breast cancer patients were identified. Of these, 158 met the inclusion criteria. Subsequently, 19 cases were excluded due to incomplete MRI, 31 for lacked adequately documented LN dissection, and 15 cases were omitted due to missing data. Consequently, 93 female breast cancer patients were deemed eligible for this study, with a median age of 56 years (range 30–89) (Fig. 4).

Among these patients, 18 (19%) underwent mastectomy, whereas 75 (81%) had a quadrantectomy. Histopathological evaluation of the surgical specimens revealed the following distribution: 3 patients were diagnosed with high-risk ductal carcinoma *in situ*, 38 with luminal A carcinoma, 19 with invasive luminal B Human Epidermal Growth Factor Receptor 2-negative carcinoma, 12 with luminal B Human Epidermal Growth Factor Receptor 2-positive, 13 with invasive Human Epidermal Growth Factor Receptor 2-enriched, and 8 with triple-negative breast cancer (Table 2).

**Table 2:** Patients’ characteristics

Variables	Center 1 (*n* = 93)	Center 2 (*n* = 18)
Age (years)^*^	56 (30–89)	48 (37−81)
Sex		
Female	93 (100.0)	18 (100.0)
Ethnicity		
Caucasian	93 (100.0)	18 (100.0)
Histologic subtypes		
Ductal carcinoma *in situ*	3 (3.2)	2 (11.1)
Luminal A	38 (40.9)	5 (27.8)
Luminal B HER2-negative	19 (20.4)	4 (22.2)
Luminal B HER2-positive	12 (12.9)	3 (16.7)
HER2-positive	13 (14.0)	2 (11.1)
Triple negative	8 (8.6)	2 (11.1)
Surgery		
Mastectomy	18 (19.4)	3 (16.7)
Quadrantectomy	75 (80.7)	15 (83.3)

Data are numbers of participants, with percentages in parentheses, except for age, which is given as mean and range (minimum−maximum)

H*ER2* Human epidermal growth factor receptor 2

These 93 MRI datasets were collected, each containing a single volume of interest corresponding to a unique subject. Of these, 40 datasets (43%) were classified as positive for LN involvement based on histopathological confirmation, while 53 datasets (57%) were classified as negative. These datasets were used for the training, validation, and testing of five machine-learning models for binary classification. Moreover, we also collected 18 samples, to form an external testing set to test the best model obtained, composed of 9 samples (50%) belonging to the positive class and 9 samples (50%) belonging to the negative class.

The selection process and demographic characteristics of the patients enrolled for external testing are detailed in Fig. 4 and summarized in Table 2.

### Radiomics-based machine-learning modeling

**Fig. 5:**
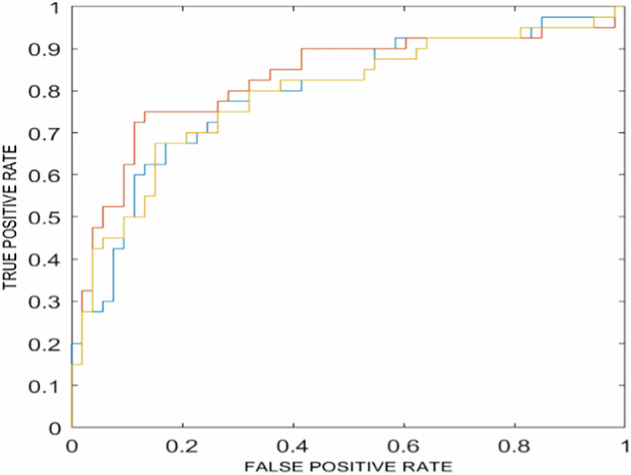
Receiver operating characteristic (ROC) curves for the best-performing ensemble of models, obtained from internal testing using aggregated predictions. The figure illustrates three ensembles of logistic regression classifiers. Each ensemble was trained within a nested k-fold cross-validation framework, in which different random splits of the data were used for training, validation, and internal testing. The curves represent the overall aggregated performance of each ensemble rather than that of individual models. The colors (red, blue, and yellow) correspond to the three ensembles; however, they do not represent fixed datasets, as each ensemble was trained on a distinct random partitioning of the data

From each segmented volume of interest, 3,380 IBSI-compliant radiomic features were extracted using the TRACE4Research™ platform. Following feature selection, 17 features were retained for model development. These features, which also resulted in stable (ICC ≥ 0.75), were used in nested 3-fold cross-validation to train, validate, and test five machine-learning classifiers for the binary task of identifying positive *versus* negative LN involvement cases. Performance metrics of the best performing model, including AUROC, accuracy, sensitivity, specificity, positive predictive value (PPV), and negative predictive value (NPV), are summarized in Table 3. Figure 5 illustrates the corresponding AUROCs: logistic regression emerged as the best-performing classifier, achieving an AUROC of 81% (95% confidence interval 78–85%), accuracy of 75% (70–79%), sensitivity of 52% (41–62%), specificity of 92% (86–98%), PPV of 85% (76–95%), and NPV of 72% (68–76%); it was therefore tested on the external testing set (9 samples, 50% belonging to positive class and 9 samples, 50%, belonging to negative class), with performances reported in Table 4.

**Table 3:** Performance metrics for the best-performing machine learning model

Model of three ensembles of logistic regression classifiers	Training	Validation	Internal testing	Internal testing (44% threshold)
AUROC	80 (76−85)	76 (71−81)	81 (78−85)	81
Accuracy	74 (71−77)	69 (66−73)	75 (70−79)	74
Sensitivity	51 (41−60)	43 (33−52)	52 (41−62)	80
Specificity	92 (89−95)	90 (86−94)	92 (86−98)	70
PPV	84 (80−89)	78 (71−85)	85 (76−95)	67
NPV	73 (69−76)	69 (66−72)	72 (68−76)	82

Data are percentages and 95% confidence intervals in parentheses

*AUROC* Area under the receiver operating characteristic curve, *NPV* Negative predictive value, *PPV* Positive predictive value

**Table 4:** Classification performance of the model of three ensembles of logistic regression classifiers for the external testing set

External testing (44% threshold)	
AUROC (%)	94 (89–99)
Accuracy (%)	94 (73–100)
Sensitivity (%)	89 (52–100)
Specificity (%)	100 (66–100)
PPV (%)	100 (63–100)
NPV (%)	90 (56–100)

Data are percentages and 95% confidence intervals in parentheses

*AUROC* Area under the receiver operating characteristic curve, *NPV* Negative predictive value, *PPV* Positive predictive value

### Statistical analysis of radiomic features

The 17 selected radiomic features and their corresponding IBSI feature families are detailed in Supplementary material (Table S1). These predictors were ranked by statistical significance, with median values, 95% confidence intervals, and results from univariate Wilcoxon rank-sum tests provided. Adjusted *p*-values for significance are also reported. Violin and box plots of these features are shown in the Supplementary material (Fig. S1), illustrating the distribution of radiomic predictors across the two classes.

### Comparison with Node-RADS

The performance comparison between the radiomics-based logistic regression model and the Node-RADS scoring system (positivity threshold > 2) was conducted using the cases included in the external validation dataset. No significant differences were observed in diagnostic accuracy (*p* = 0.083), sensitivity (*p* = 0.157), or specificity (*p* = 0.317). Cohen's κ coefficient was 0.47, indicating moderate agreement between the two approaches. The radiomics model demonstrated enhanced specificity (100% *versus* 89%) compared to Node-RADS (Table 5).

**Table 5:** Results of the McNemar test comparing the performance of the radiomics-based logistic regression model to the Node-RADS

Metric	Radiomic-based logistic regression model			Node-RADS		
	Ratio	Point estimate	95% CI	Ratio	Point estimate	95% CI
Sensitivity	8/9	89	52–100	6/9	68	30–92
Specificity	9/9	100	66–100	8/9	89	52–99
Accuracy	17/18	94	73–100	14/18	78	52–94

Data are percentages and 95% confidence interval

## Discussion

This proof-of-concept study suggests that radiomics-based machine-learning models using breast MRI data may offer comparable diagnostic performance to the Node-RADS system in a relatively small cohort of patients. The logistic regression model, which achieved the highest AUROC (81%), showed high specificity (92%) and PPV (85%), positioning it as a promising noninvasive decision-support tool. While its moderate sensitivity (52%) limits the detection of all positive cases, the model’s high specificity is particularly valuable in borderline patients, reducing unnecessary surgical interventions and guiding more accurate diagnostic evaluations. The comprehensive radiomic feature analysis supports its utility in capturing subtle imaging biomarkers associated with LN involvement.

While the radiomics-based logistic regression model demonstrates high specificity and PPV, its overall accuracy and sensitivity were not significantly different from those achieved with the Node-RADS scoring system, a straightforward method. Compared to Node-RADS, radiomics involves additional complexities, such as manual segmentation, the need for computational resources, and specialized technical expertise, which currently limit its widespread adoption. In the small cohort of the external validation, Node-RADS demonstrated comparable diagnostic performance, maintaining moderate agreement with the radiomics-based model (Cohen κ = 0.47).

Although radiomics does not yet provide a definitive advantage in standard breast cancer management, it holds potential as a complementary tool in specific contexts, such as cases with limited operator expertise or resource constraints for implementing Node-RADS. Radiomics also offers opportunities to identify imaging biomarkers that reflect tumor biology, a promising avenue for future applications in risk stratification and treatment monitoring.

The integration of radiomics-based machine learning models for predicting axillary LN involvement in breast cancer aligns well with advancements in imaging-driven diagnostics across oncology [6]. Similar studies have explored various imaging modalities, such as positron emission tomography/computed tomography [17], ultrasound [18], and MRI [5, 8], emphasizing their potential for accurate, noninvasive LN involvement assessment. For instance, positron emission tomography/computed tomography-based radiomic models achieved AUROC values up to 88% while showing strengths in sensitivity [17]. Ultrasound, combined with radiomics, has demonstrated its capability to predict sentinel LN metastasis, particularly in clinically node-negative patients [18].

In the context of breast MRI, prior research integrating radiomics and deep learning achieved AUROC values ranging from 80% to 88%, reinforcing the potential of MRI as a valuable modality for axillary assessment [8]. Furthermore, the introduction of Node-RADS has provided a standardized scoring system for MRI-based LN evaluation, showing moderate-to-high diagnostic accuracy and excellent inter-reader agreement (Cohen κ = 0.72–0.83) [4, 5]. This study’s findings, particularly the high specificity and PPV of the radiomics-based logistic regression model, are consistent with these outcomes, confirming the utility of MRI radiomics as a complementary tool. Notably, this study emphasizes the robustness of the TRACE4Research™ platform, which adheres to IBSI-compliant workflows for radiomic feature extraction [14]. In addition to its diagnostic potential, the use of radiomics offers a pathway for exploring imaging biomarkers that may correlate with treatment response or prognosis [6, 19]. The ability to quantitatively assess LN characteristics noninvasively may provide insights into tumor biology, aiding in longitudinal monitoring and therapy optimization [19, 20].

The retrospective design and modest sample size (93 cases) provide a strong foundation for proof-of-concept validation, though larger, diverse cohorts are needed to enhance generalizability. While the logistic regression model’s sensitivity is moderate, integration with clinical or molecular data could improve performance. Manual segmentation, usually performed in similar studies, introduces some variability, which future automation could address to enhance reproducibility and streamline workflows [6, 21].

The clinical implications of this study are relevant. By demonstrating comparable performance to the Node-RADS scoring system, the radiomics-based machine-learning model offers a reproducible, noninvasive alternative for LN involvement assessment. High specificity (92%) and PPV (85%) suggest that this approach could effectively reduce false-positive diagnoses, minimizing unnecessary invasive procedures such as sentinel LN biopsy [2, 9] or axillary LN dissection [3, 9].

In fact, axillary de-escalation surgery is becoming a key strategy in breast cancer management to reduce surgical morbidity while ensuring oncological safety. The transition from routine axillary LN dissection to less invasive approaches, such as sentinel LN biopsy or omission of axillary surgery in selected cases, depends on accurate preoperative LN assessment. The proposed radiomics-based model could aid in patient selection, improving specificity and PPV.

However, the model’s moderate sensitivity (52%) indicates that it cannot replace surgical staging and should be used as a complementary tool rather than a standalone method for LN assessment. Its integration with established imaging-based classification systems, such as Node-RADS, may enhance preoperative risk stratification and improve decision-making in borderline cases. Moreover, the integration of radiomics into clinical workflows aligns with the broader goal of precision medicine [6], allowing for tailored treatment strategies based on individual risk profiles [22]. For example, patients with a high probability of LN involvement could proceed directly to appropriate systemic therapy or surgical planning, while those with a low probability might avoid unnecessary interventions.

One relevant limitation of this proof-of-concept study is related to the very small sample size for the external validation (only 18 patients), limiting the robustness/generalizability of the findings and the statistical power for comparisons. In addition, the use of a single expert reader for Node-RADS scoring may further reduce generalizability. However, the encouraging performance observed for the radiomics-based approach (AUROC 94%) suggests potential utility for these models in non-invasive LN assessment, warranting further validation in larger, prospective multi-center studies to mitigate possible selection bias or overfitting effects.

The study’s findings suggest several directions for future research. Refining feature selection or integrating multimodal data, such as radiomic features from other imaging modalities, clinical factors, and molecular biomarkers, could improve predictive performance [8, 19]. Advanced machine learning methods, including ensemble techniques and deep learning frameworks like convolutional neural networks or vision transformers, may also enhance accuracy [17, 23, 24]. Developing fully automated workflows for segmentation and feature extraction would reduce inter-observer variability and improve practicality in clinical settings. Finally, large-scale, multicenter validation studies are needed to standardize protocols and ensure reproducibility. Exploring the prognostic potential of radiomic biomarkers could further support decisions on neoadjuvant therapies and long-term follow-up [19, 22, 24].

In conclusion, this study explored the role of radiomics-based machine-learning models as decision-support tools in breast cancer management. In this proof-of-concept study, a radiomics-based machine-learning model showed an interesting diagnostic performance (AUROC 94%) with accuracy comparable to Node-RADS. However, its current complexity—including manual segmentation, feature extraction, and model implementation—limits its applicability in routine clinical practice. It may offer complementary diagnostic value in selected scenarios, such as borderline cases or settings with limited radiological expertise. Further validation in larger prospective studies is warranted [25].

## Supplementary information

**Additional file 1: Table S1.** Ensemble of logistic regression classifiers. **Fig. S1.** Violin and box plots of the I-XVII radiomic predictors. Violin and box plots of “positive” and “negative” classes are reported in red and green, respectively.

## Data Availability

The datasets generated and analyzed during the current study are not publicly available due to institutional restrictions and patient confidentiality policies. However, de-identified data and radiomic feature sets may be shared upon reasonable request to the corresponding author, subject to institutional approval. The code used for radiomic feature extraction and modeling is available upon request for reproducibility purposes.

## Abbreviations

AUROC: Area under the receiver operating characteristic curve
IBSI: Image biomarker standardization initiative
ICC: Intraclass correlation coefficient
LN: Lymph node
MRI: Magnetic resonance imaging
NPV: Negative predictive value
PPV: Positive predictive value
