# Supplementary material for: Radiomics-based MRI models for predicting breast cancer axillary lymph node involvement in comparison with Node-RADS: a proof-of-concept study
Source: Eur Radiol Exp. 2025 Dec 2;9:117. doi: 10.1186/s41747-025-00660-4 (PMC12672992; doi:10.1186/s41747-025-00660-4)
Supplement: Supplementary file 1 — Additional file 1: Table S1. Ensemble of logistic regression classifiers. Fig. S1. Violin and box plots of the I-XVII radiomic predictors. Violin and box plots of “positive” and “negative” classes are reported in red and green, respectively. [file 41747_2025_660_MOESM1_ESM.pdf]

Radiomics-based MRI models for predicting breast cancer axillary lymph node involvement in comparison with Node-RADS: a proof-of-concept study

ELECTRONIC SUPPLEMENTARY MATERIAL

Table S1 Ensemble of logistic regression classifiers

#	Feature family	Feature nomenclature	Median in the positive class (95% CI)	Median in the negative class (95% CI)	Uncorrected *p*-value	Corrected *p*-value
I	Neighbouring Grey Level Dependence Matrix	Subtraction - Logarithm Grey Level Non Uniformity	2.29e+02 (1.62e+02 - 2.96e+02)	9.11e+01 (7.33e+01 - 1.09e+02)	< 0.05	< 0.05
II	Neighbouring Grey Level Dependence Matrix	Subtraction - Square Dependence Count Non Uniformity	3.80e+02 (2.35e+02 - 5.24e+02)	1.66e+02 (1.34e+02 - 1.97e+02)	< 0.05	< 0.05
III	Neighbouring Grey Level Dependence Matrix	Subtraction - Squareroot Low Dependence Emphasis	3.89e-01 (3.64e-01 - 4.15e-01)	4.77e-01 (4.57e-01 - 4.97e-01)	< 0.05	< 0.05
IV	Neighbouring Grey Level Dependence Matrix	T2W - Wavelet LLH Dependence Count Energy	1.36e-02 (1.26e-02 - 1.46e-02)	1.59e-02 (1.52e-02 - 1.65e-02)	< 0.05	< 0.05
V	Neighbourhood Grey Tone Difference Matrix	Subtraction - Wavelet HHL Strength	1.57e+00 (1.05e+00 - 2.10e+00)	3.32e+00 (2.82e+00 - 3.81e+00)	< 0.05	< 0.05
VI	Neighbourhood Grey Tone Difference Matrix	Subtraction - Wavelet LHH Strength	1.63e+00 (1.20e+00 - 2.05e+00)	3.25e+00 (2.70e+00 - 3.80e+00)	< 0.05	< 0.05
VII	Grey-Level Co-Occurrence Matrix	Subtraction - Squareroot Inverse Difference Moment	1.79e-01 (1.63e-01 - 1.95e-01)	1.49e-01 (1.42e-01 - 1.56e-01)	< 0.05	< 0.05
VIII	Grey-Level Co-Occurrence Matrix	Subtraction - Squareroot Inverse Difference	2.64e-01 (2.47e-01 - 2.82e-01)	2.34e-01 (2.26e-01 - 2.42e-01)	< 0.05	< 0.05
IX	Neighbourhood Grey Tone Difference Matrix	Subtraction - LoG Busyness	4.68e-02 (3.44e-02 - 5.92e-02)	2.69e-02 (2.30e-02 - 3.07e-02)	< 0.05	< 0.05
X	Grey-Level Co-Occurrence Matrix	T2W - Square Correlation	5.76e-01 (5.26e-01 - 6.26e-01)	4.74e-01 (4.38e-01 - 5.11e-01)	< 0.05	< 0.05
XI	Grey-Level Run Length Matrix	Subtraction - Wavelet LHL Grey Level Variance	5.07e+01 (4.61e+01 - 5.53e+01)	6.55e+01 (5.88e+01 - 7.21e+01)	< 0.05	< 0.05
XII	Intensity Histogram	Subtraction - Wavelet LHL Variance	4.97e+01 (4.52e+01 - 5.42e+01)	6.35e+01 (5.67e+01 - 7.03e+01)	< 0.05	< 0.05
XIII	Grey-Level Size Zone Matrix	T2W - Squareroot Grey Level Non Uniformity Normalized	2.48e-02 (2.30e-02 - 2.67e-02)	2.86e-02 (2.74e-02 - 2.97e-02)	< 0.05	< 0.05
XIV	Grey-Level Size Zone Matrix	Subtraction - Square Zone Size Non Uniformity Normalized	4.21e-01 (4.04e-01 - 4.37e-01)	4.54e-01 (4.42e-01 - 4.66e-01)	< 0.05	< 0.05
XV	Intensity Histogram	T2W - Squareroot 90th Percentile	4.20e+01 (3.88e+01 - 4.52e+01)	3.90e+01 (3.66e+01 - 4.14e+01)	< 0.05	0.35
XVI	Grey-Level Co-Occurrence Matrix	T2W - Exponential Sum Average	1.06e+01 (9.17e+00 - 1.21e+01)	9.82e+00 (8.67e+00 - 1.10e+01)	0.11	1.00
XVII	Neighbourhood Grey Tone Difference Matrix	T2W - Wavelet HLH Complexity	4.21e+03 (3.76e+03 - 4.66e+03)	4.31e+03 (3.69e+03 - 4.94e+03)	0.88	1.00

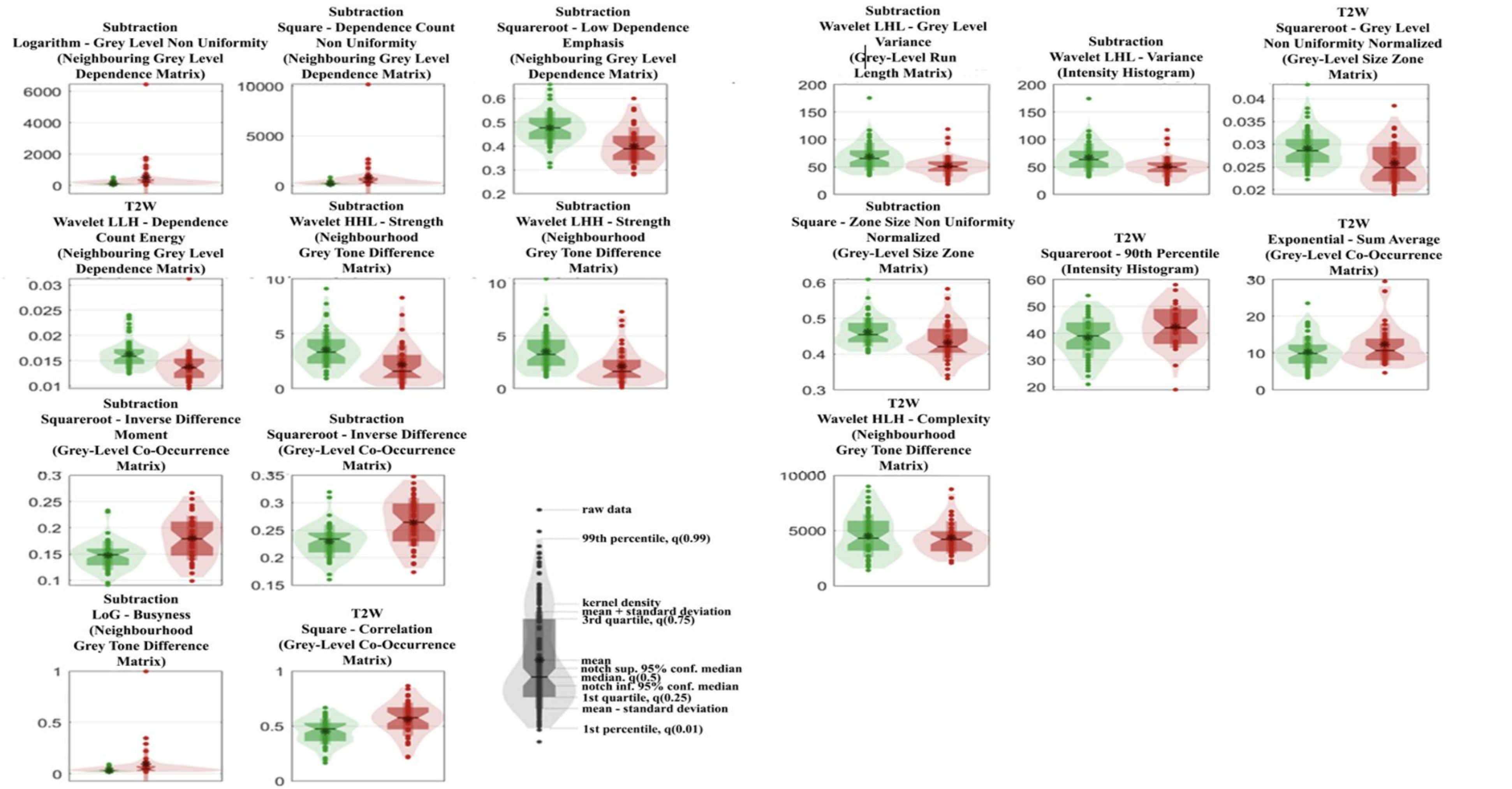

Fig. S1 Violin and box plots of the I-XVII radiomic predictors. Violin and box plots of "positive" and "negative" classes are reported in red and green, respectively.
